# Sometimes memory misleads: Variants of the error-speed effect strengthen the evidence for systematically misleading memory signals in recognition memory

**DOI:** 10.3758/s13423-024-02534-z

**Published:** 2024-07-01

**Authors:** Anne Voormann, Annelie Rothe-Wulf, Constantin G. Meyer-Grant, Karl Christoph Klauer

**Affiliations:** Department of Psychology, University of Freiburg, Freiburg, Germany

**Keywords:** Recognition memory, Confidence-rating tasks, Binary recognition task, Misleading memory evidence

## Abstract

The error-speed effect describes the observation that the speed of recognition errors in a first binary recognition task predicts the response accuracy in a subsequent two-alternative forced-choice (2AFC) task that comprises the erroneously judged items of the first task. So far, the effect has been primarily explained by the assumption that some error responses result from misleading memory evidence. However, it is also possible that the effect arises because participants remember and use their response times from the binary task to solve the 2AFC task. Furthermore, the phenomenon is quite new and its robustness or generalizability across other recognition tasks (e.g., a confidence-rating task) remains to be demonstrated. The aim of the present study is to address these limitations by introducing a new variant of the error-speed effect, replacing the 2AFC task with a confidence-rating task (Experiment 1), and by reversing task order (Experiment 2) to test whether participants employ a response-time strategy. In both experiments, we collected data using a sequential probability ratio *t*-test procedure and found evidence in favor of the hypothesis that the speed of binary recognition errors predicts confidence ratings for the same stimulus. These results attest to the robustness and generalizability of the error-speed effect and reveal that at least some errors must be due to systematically misleading memory evidence.

## Introduction

The error-speed effect refers to a relatively new phenomenon in the recognition-memory literature that has been interpreted primarily as evidence for the existence of systematically misleading memory evidence, namely memory evidence in favor of the incorrect response (Starns et al., 2018). The effect arises when participants are asked to complete both a binary and a two-alternative forced-choice (2AFC) recognition task comprising the same stimuli. In a binary recognition task, participants have to categorize presented stimuli either as target (i.e., a stimulus that has been studied previously) or as lure (i.e., a completely new stimulus). In a 2AFC task, on the other hand, a pair of stimuli is presented in each trial – a target and a lure – and participants have to identify the target. When those two tasks are completed in succession, it has been observed that the speed of errors in a binary recognition task predicts the recognition accuracy in a subsequent 2AFC trial containing the erroneously judged stimulus of the first task. More precisely, fast errors in the binary recognition task are associated with lower accuracy in the 2AFC task than slow errors depicting the error-speed effect (Starns et al., 2018; Voormann et al., 2021, 2024).

Theoretical accounts allowing for incorrect responses to targets and lures based on misleading memory evidence typically predict an error-speed effect. For example, continuous dynamic recognition models, such as the diffusion model (Ratcliff, 1978), postulate that the speed of recognition decisions depends on the strength of the underlying memory signal, with more decisive memory signals eliciting faster responses (Ratcliff, 2014).^1^ This holds for both correct and incorrect responses. The same principle applies to the accuracy of responses. Here, for example, a strongly misleading memory signal increases the probability of an incorrect response (Ratcliff et al., 2015). Considering the two above-mentioned recognition tasks performed in sequence, this implies that the more misleading the memory signal elicited by a stimulus, the faster a stimulus is erroneously categorized in the binary recognition task and the more likely is an incorrect response given to a trial of the 2AFC task that includes the same stimulus.

**Fig. 1:**
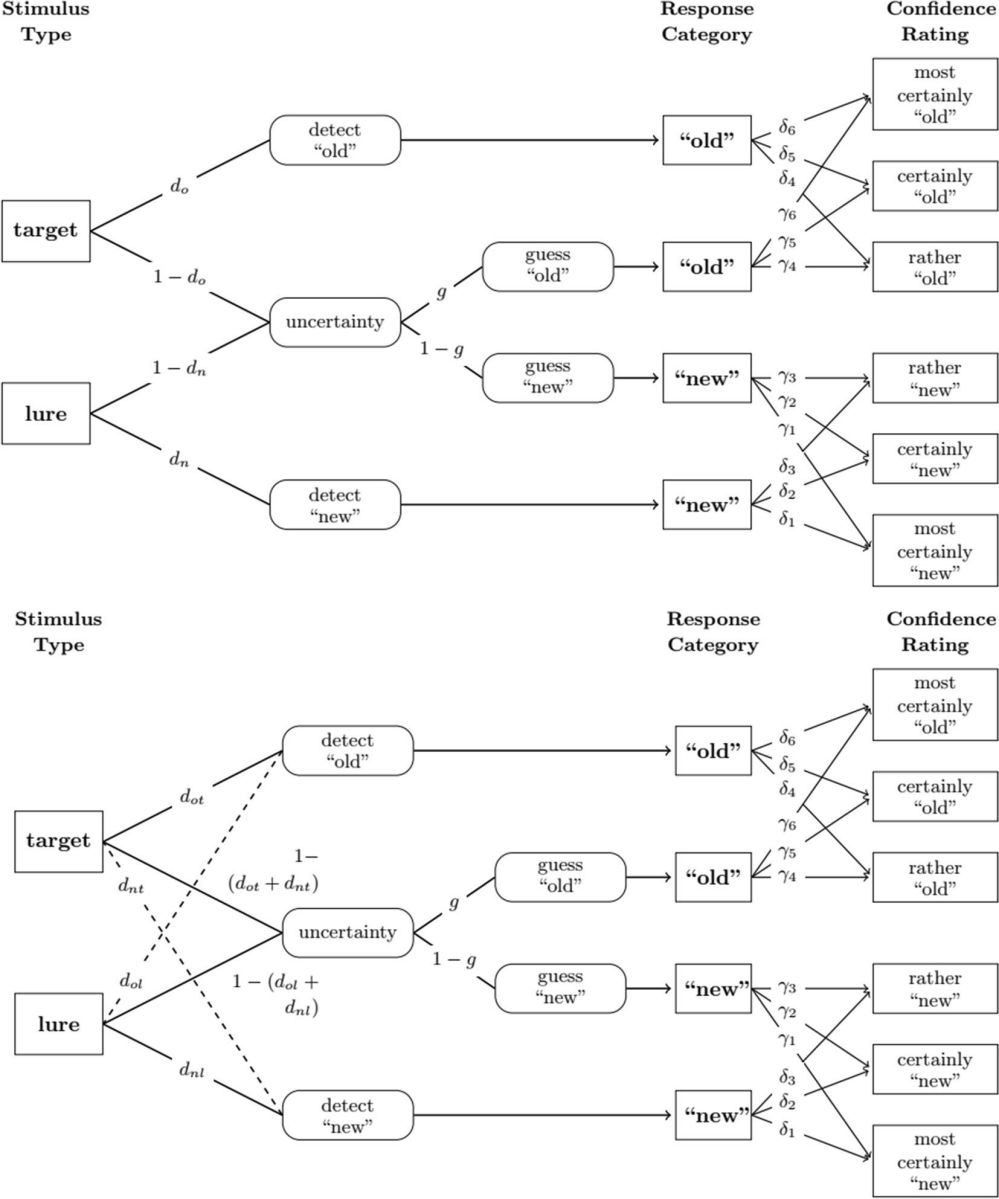
Depiction of the two-high threshold-model (upper panel) and the two-low threshold-model (lower panel) for a recognition task including confidence ratings. *Note.* The models represent the paths to certain response categories and confidence ratings for the two stimulus types (i.e., target and lure). Discrete latent states are depicted in rounded boxes with the conditional probability to enter each state denoted on the respective path. Dashed lines indicate the differences between the two-high and the two-low threshold-model

Besides continuous models, some discrete-state models of recognition memory that incorporate a pathway towards incorrect detection, such as the two-low threshold-model (2LTM; Starns, 2021; Starns et al., 2018; see Fig. 1), also predict the error-speed effect. Following the core idea behind response-time-extended multinomial processing tree models (Klauer & Kellen, 2018), response speed is determined by the sum of process times associated with different discrete states that contribute to a response. Thus, assuming that responses out of an incorrect detection state are faster than incorrect guesses (Heck & Erdfelder, 2016; Province & Rouder, 2012), faster errors should go along with a higher probability of an incorrect response in a subsequent 2AFC task. However, discrete-state models that do not incorporate the possibility of incorrect detection, such as the two-high threshold-model (2HTM; Snodgrass & Corwin, 1988), cannot predict this pattern of results. This is because of assumed complete loss of information when entering a discrete state (Riefer & Batchelder, 1988). In the latter model, errors occur solely due to incorrect guessing decisions, and thus, the speed of binary recognition errors should not affect response accuracy since all errors reflect the same underlying process (see Fig. 1). Based on this theoretical rationale, the error-speed effect has mostly been considered as evidence for the existence of misleading memory evidence.

However, an alternative explanation for the error-speed effect was already discussed in the original manuscript by Starns et al. (2018), namely that participants notice their previous response times (RTs) from the binary task and use their knowledge about them to inform their decisions in the 2AFC task. For example, in cases where both items of the 2AFC task elicit an “old” response, participants might remember the item with the faster RT in the binary recognition task and then select this item as target in the 2AFC task. Although previous studies of the error-speed effect ruled out some alternative explanations (Voormann et al., 2021, 2024), none of them explicitly tested the RT-strategy hypothesis as all prior studies asked for responses in the binary recognition task first and only afterwards presented the 2AFC task. Recently, Akan et al. (2023) and Yüvrük et al. (2023) observed an error-speed effect for targets in a trial-by-trial paradigm in which each 2AFC trial presented an item from the preceding trial of the binary task along with an item not previously considered during test. This rules out certain versions of the RT-strategy hypothesis capitalizing on a comparison between the RTs associated with previous responses to the two presented items, but leaves open the possibility of RT strategies based on the RT of the (one) item previously responded to. For example, participants might still prefer to choose the item they remember having responded to fast in order to resolve uncertainties in the 2AFC task.

In the present study, we therefore not only aimed to replicate the error-speed effect using confidence ratings in Experiment 1 to demonstrate its independence of the specific combination of tasks, but we also reversed task order in Experiment 2; that is, we presented the confidence-rating task first and the binary recognition task afterwards. In such a case, participants can no longer rely on their knowledge about the response speed in the binary task when giving their confidence ratings, as binary responses are given only after the confidence-rating task. Thus, if reversing task order eliminates the error-speed effect, it is quite likely that its existence is rooted in some kind of RT-based strategy. However, if the error-speed effect still occurs with reversed task order it seems more likely that the error-speed effect is indeed driven by misleading memory evidence.

## The error-speed effect with confidence-rating tasks

Asking for confidence ratings is a very popular recognition memory task (e.g., Dube et al., 2012; Province & Rouder, 2012; Ratcliff et al., 1994). In such a task, participants indicate how certain they are that a presented stimulus is a target (or a lure) on a scale that could, for instance, range from “most certainly new” to “most certainly old,” and different responses are interpreted to reflect variability in the underlying memory evidence. Previous studies have demonstrated that when confidence ratings are provided immediately after a binary decision, confidence ratings and RTs in binary tasks are correlated (e.g., Weidemann & Kahana, 2016). Therefore, confidence-rating tasks seem a natural choice if one seeks to replace the original 2AFC task.

**Fig. 2:**
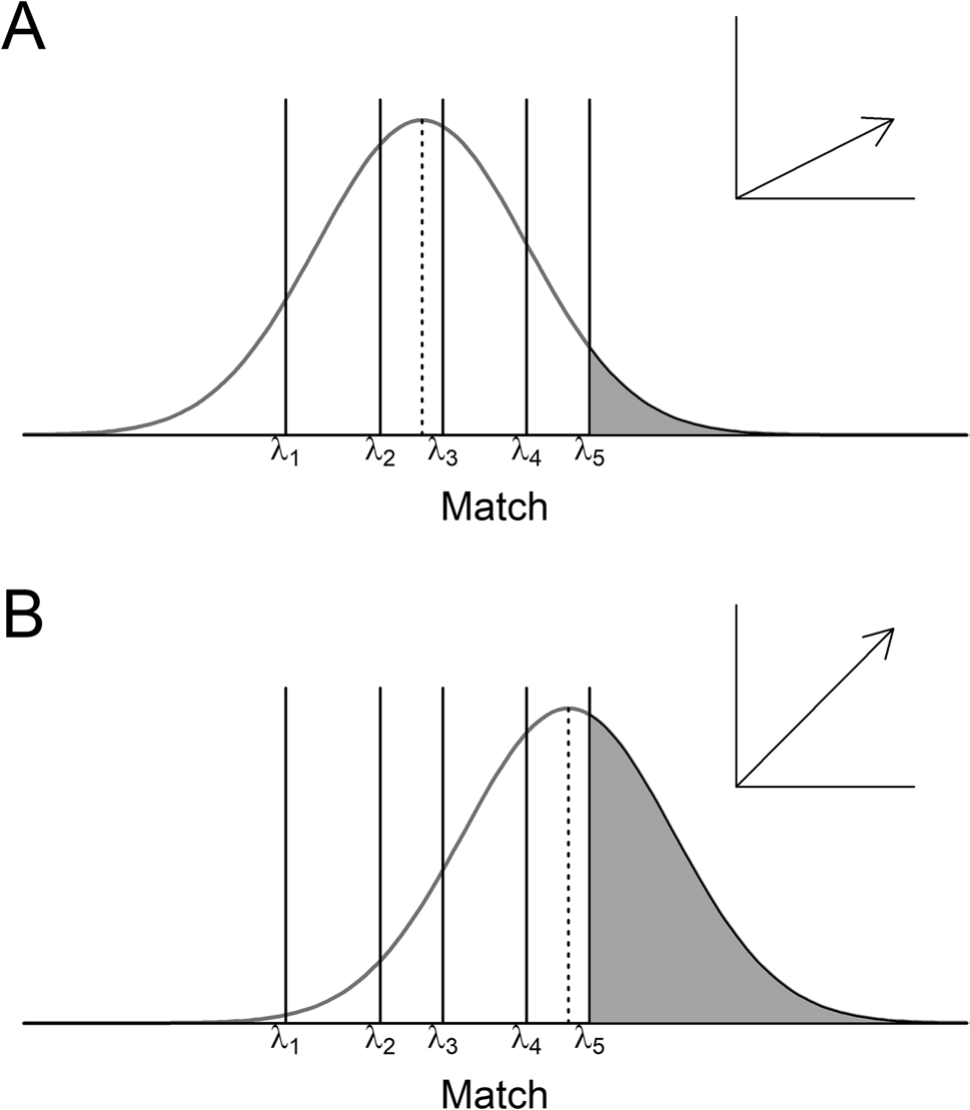
Depiction of the RTCON2 model for an item eliciting low memory evidence (panel A) and an item with high memory evidence (panel B). *Note.* Vertical lines represent the position of the confidence criteria whereas the drift rate of the accumulator for a specific confidence response is determined by the area under the curve of the corresponding confidence interval, illustrated in the figure by the grey area for the “most certainly old”-response

When replacing the 2AFC task in the error-speed paradigm with a confidence-rating task, one would expect to find a pattern of effects that is analogous to the error-speed effect. Whereas for the RTs of the binary recognition task the principles of the classical diffusion model still apply, this model is not capable of taking confidence ratings into account. However, there is an adapted version of the diffusion model for confidence ratings known as RTCON2 (Ratcliff & Starns, 2013; see also Ratcliff & Starns, 2009, for a previous version of the model). Under the assumptions of RTCON2, each confidence response has its own accumulation process with response boundaries that can differ across accumulators but are constant over trials (Ratcliff & Starns, 2013). More importantly, the accumulation rate of each accumulator depends on the memory evidence elicited by a specific stimulus. However, in contrast to binary tasks, in which memory evidence is often considered to be a single fixed value, each stimulus gives rise to a distribution on an underlying strength-of-evidence scale: the so-called match distribution (see Fig. 2). Response criteria are placed on that scale demarcating confidence intervals. The area under the curve of this match distribution in a certain confidence interval in turn defines the accumulation rate for the associated accumulator. Therefore, a target with a very high memory evidence should elicit a match distribution that is shifted towards a “most certainly old” response, which increases the accumulation rate (and consequently the probability) for a “most certainly old” response (Fig. 2B) compared to a target with a low memory evidence (Fig. 2A). On the other hand, a target with low memory evidence would elicit a match distribution that allocates more probability mass towards a new response and will therefore more likely lead to an erroneous “rather new” response.

Under the assumption of stable response criteria and constant boundaries for the accumulation process across trials, it follows that the more the match distribution of a certain item is shifted towards the low or high end of the latent evidence scale, the higher the likelihood of confidence ratings in the corresponding direction for that item (Ratcliff & Starns, 2013). Returning to the basis of the error-speed effect, this means that the faster an erroneous response in the binary recognition task, the more misleading its memory evidence (based on the predictions of the diffusion model). According to the RTCON2, this should lead to a higher confidence towards the incorrect response (cf. Van Zandt, 2000, for another dynamic confidence model that predicts the same outcome).

There also exist extensions of discrete-state models that allow one to account for confidence ratings. Here, different confidence responses arise from the final discrete state reached in each case (Bröder et al., 2013; see also Klauer & Kellen, 2010). Again, such responses are independent of the underlying amount of memory evidence in discrete-state models. Instead, they are only contingent on the discrete state they result from (see Fig. 1). Crucially, however, the likelihood of entering the different discrete states depends on the memory evidence elicited by a stimulus. This leads to analogous predictions as for the classic error-speed effect: Only models that include the possibility for incorrect detection (such as the 2LTM) can explain a correlation between the response speed in a first recognition task and the confidence response given in a second recognition task. Importantly, those models consider two different mental states that can result in an erroneous response (viz. an uncertainty state and an incorrect detection state), and the respective pathways may contribute in different proportions to fast and slow errors. However, models that do not allow for incorrect detection states and instead assume that all errors stem from a single uncertainty state (as, e.g., the 2HTM) cannot predict a relationship between response speed in a first recognition task and confidence in a second recognition task (but see Voormann et al., 2024).

As the reviewed theories predict an effect similar to the error-speed effect when replacing the 2AFC task by a confidence-rating task, our first experiment tests whether the speed of erroneous recognition decisions in a binary recognition task predicts the confidence given to that same stimulus in a subsequent confidence-rating task. More precisely, we expect fast-error stimuli to be associated with, on average, more confidence towards the incorrect response compared to slow-error stimuli. If so, it seems more likely that the error-speed effect reflects core cognitive mechanisms rather than being a mere byproduct of using a specific combination of tasks. This would challenge models such as the 2HTM that do not allow for systematically misleading memory evidence, and would endorse models that allow for such evidence.

## The error-speed effect with reversed task order

Replicating the error-speed effect with confidence ratings is a necessary precondition for the aim of our Experiment 2, namely to conduct a direct test of the aforementioned RT strategy by reversing task order. If we assume that misleading evidence is the source of the error-speed effect, as explained above, one critical assumption to account for the effect is that the memory evidence for one stimulus remains approximately constant across different test occasions. As long as this precondition is met, the error-speed effect should not be contingent on the order in which participants encounter the two tasks.

For example, according to the RTCON2/diffusion model, both the speed in a binary recognition task and the confidence of the response depend on the elicited memory signal. Thus, the speed of binary recognition responses should be predictive of a later confidence judgment. At the same time, however, the confidence indicated in a first recognition task should also correlate with the response speed in a subsequent binary recognition task.

The same rationale holds for those discrete-state models that can account for the error-speed effect (e.g., the 2LTM). Binary recognition responses should correlate with the respective confidence recognition responses as the memory evidence of a stimulus should – in most cases – elicit the same discrete state in the binary task from which the confidence decisions arise (e.g., incorrect detection or guessing out of uncertainty in case of errors). This prediction holds irrespective of whether the binary or the confidence-rating task are presented first.

In contrast, a simple RT strategy in which confidence responses are based on the speed of the response given in the preceding binary recognition task is thwarted when participants provide confidence ratings prior to the binary recognition responses. Thus, in our second experiment we aim to investigate whether the effect still occurs when the order of the binary recognition task and confidence-rating task is reversed. More precisely, we predict that if the error-speed effect is indeed caused by misleading memory evidence, a higher confidence towards the incorrect response should go along with on average a faster erroneous decision in a subsequent binary recognition task. On the other hand, a failure to observe such an effect would suggest that participants use an RT-based strategy for selecting their confidence rating.

## Methods

Because Experiment 1 and Experiment 2 differ only in the order of their test blocks, we present methods and results conjointly, highlighting differences where present. Both experiments were pre-registered on the Open Science Framework (OSF) prior to data collection (see https://osf.io/3qzwh/).

### Sample

Both experiments implemented a sequential sampling plan using a sequential probability ratio *t*-tests (SPRTs), which is typically more efficient for reaching a conclusion with pre-determined power than classical test regimes with fixed sample size (Schnuerch & Erdfelder, 2020; see also Wald, 1945). The sequential test conforms to an a priori specified power (1-*β*) and *α*-error given a targeted effect size (*d*_*z*_). After every complete and valid data set, the size of the empirical likelihood ratio (LR) is compared to two criteria. The LR is the ratio of the likelihood of the observed data given the alternative hypothesis and the likelihood of the observed data under the null hypothesis (for more details see Appendix A). The *t*-distribution for the alternative hypothesis is non-central, with a non-centrality parameter corresponding to an expected effect size of *d*_*z*_. For the present study, we chose an effect size of *d*_*z*_ = 0.3 as it represented the lower boundary of the 90% confidence interval when estimating a meta effect size over the results from our previous study on error-speed effects (Voormann et al., 2021).

To calculate the inference criteria, we specified *α* = .05 and 1-*β* = .95. This led to the decision criteria *A* = 19 and *B* = .053. As long as the LR was in between the two decision criteria, sampling was continued.^2^ As pre-registered, inference criteria were checked at least once a day. If the decision boundaries had been crossed during a day while further data sets were obtained (which was the case in Experiment 1), the inference decision was based on the data sets until the first crossing of one of the decision boundaries.

Across both experiments, a total of 66 participants were tested. One participant had to be excluded due to technical problems and one participant missed the pre-registered inclusion criterion defined as a minimum difference between hit and false alarm rates of .1. This resulted in a final sample size of 48 participants in Experiment 1 (35 females, 13 males, with age ranging between 19 and 39 years, *M* = 24.1 years, *SD* = 4.9 years) and 16 participants in Experiment 2 (13 females, three males, with age ranging between 21 and 41 years, *M* = 25.4 years, *SD* = 5.1 years).

All participants spoke German fluently and had normal or corrected-to-normal vision. For complete participation, psychology students received partial course credit. All other participants received a monetary compensation instead.

### Material

As in Voormann et al. (2021), a list of 639 German nouns (taken from Lahl et al., 2009) served as word pool from which targets and lures were drawn for use in the recognition paradigms. All words were neutral in valence, between four and eight characters long and approximately equally frequent in spoken language.

### Procedure

**Fig. 3:**
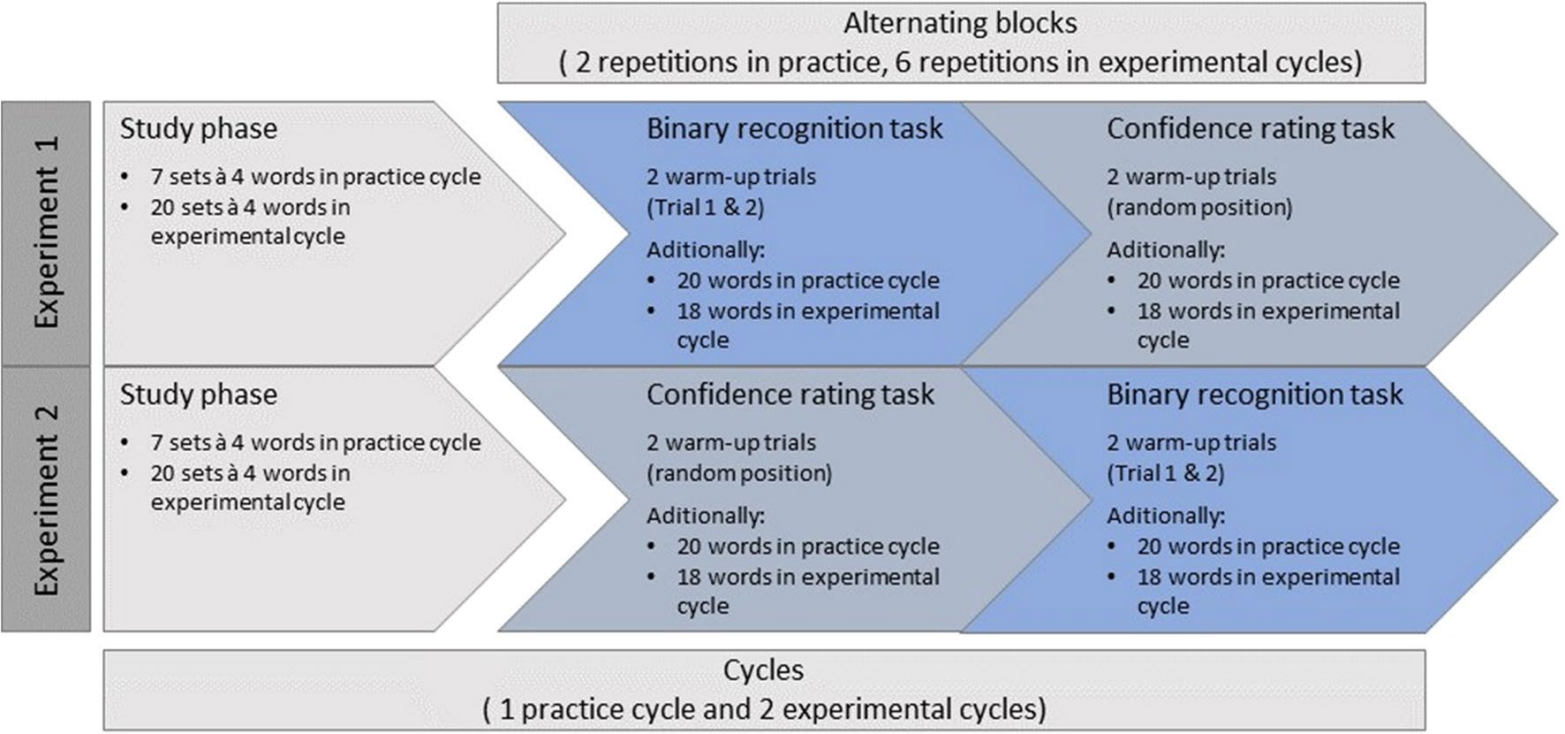
Experimental procedure of Experiment 1 and 2

We kept the procedure as close as possible to the extended condition in Voormann et al. (2021). In both experiments, participants first completed a study phase that was followed by a test phase consisting of alternating blocks of a binary recognition task and a confidence-rating task. However, the order of the tasks during the test phase differed between experiments (see Fig. 3). In total, participants completed three study-test cycles: one practice cycle, to get acquainted with the procedure, followed by two experimental cycles. The practice cycle and the experimental cycles differed in the number of trials but were identical in all other regards. Data obtained in the practice cycle were excluded from subsequent analyses.

The study phase of each cycle consisted of multiple sets of four words. The practice cycle comprised a total of seven sets (28 words), whereas each of the experimental cycles included a total of 20 sets (80 words). Participants were instructed to memorize each of the presented words to the best of their ability. The words appeared consecutively, centered on the screen, for 1,900 ms with a 100-ms blank screen separating words of the same set. After each set of four words, participants were asked to recall one specific word indicated by its position within the set. Word position was randomly chosen. If a typed response was incorrect, an error message appeared for 1,000 ms. Words from to-be recalled positions were not presented in the test phase. Furthermore, the first and the last study set served as filler sets to buffer primacy and recency effects. These sets were discarded for the analyses. However, the six words from the filler sets not recalled during study formed a separate word pool, which was used in warm-up trials in the subsequent tasks.

The study phase was followed by the test phase, which comprised alternating blocks of the binary recognition task and the confidence-rating task. In the practice cycle, participants responded to two series of these alternating task blocks. In each of the two experimental cycles, participants accomplished six series of these alternating task blocks. In Experiment 1, each series started with the binary recognition block followed by the confidence-rating block, whereas in Experiment 2, each series started with the confidence-rating block followed by the binary recognition block.^3^

In the binary recognition task, participants were asked to indicate as quickly and as accurately as possible whether a presented word was new (not studied in the preceding study phase) or old (studied in the study phase). Words were presented individually in the center of the screen until a response was recorded. To remind participants of the instructed response mapping, the words “ALT” and “NEU” (German for “OLD” and “NEW”) were presented below the stimulus paired with the respective response keys (“Y” for new words and “-” for old words on a German QWERTZ keyboard).

In the confidence-rating task, participants were asked to indicate their level of confidence that a word had or had not been previously studied. Participants rated each word on a scale from 1 “very sure NEW” to 6 “very sure OLD.” Words were again presented individually in the center of the screen until a response was given. As in the binary recognition task, the response mapping appeared on the screen below the stimulus depicting a rating scale aligned from left to right (‘1’ – “Sehr sicher NEU” [“most certainly NEW”]; ‘2’ – “Sicher NEU” [“certainly NEW”]; ‘3’ – “Eher NEU” [“rather NEW”]; ‘4’ – “Eher ALT” [“rather OLD”]; ‘5’ – “Sicher ALT” [“certainly OLD”]; ‘6’ – “Sehr sicher ALT” [“most certainly OLD”]). To provide their answer, participants used the number keys from 1 to 6 above the letter keys on a German QWERTZ keyboard. Once a response was entered, a red cross appeared for 500 ms at the position of the corresponding number. After a blank screen of 50 ms, the next stimulus followed.

Targets and lures appeared equally frequent in each block, with lures being randomly drawn from the remaining word pool. In the practice cycle, each block included 20 words (ten targets and ten lures); in the two experimental cycles, each block consisted of 18 words (nine targets and nine lures). Additionally, two warm-up trials consisting of a target and a lure were presented at the beginning of each binary recognition block to accommodate for slower response times usually observed in the first trials of a block. The target presented in those warm-up trials was randomly drawn from the pool of not-to-be recalled words of the filler blocks in the respective study phase, while the lure was randomly sampled from the remaining words in the word pool.

To maintain a constant block size between binary recognition blocks and confidence-rating blocks, the two warm-up trials were also included in the confidence-rating task. However, these trials were distributed randomly within the block and were excluded from further analyses. For each series of alternating blocks, the set of words presented in each binary recognition block matched the words of the corresponding confidence-rating block.

## Analysis for the sequential probability ratio *t*-tests (SPRTs)

For both experiments, the sequential sampling procedure was based on the value of the *t* statistic from a *t*-test comparison of the mean confidence ratings for fast versus slow errors observed in the binary recognition task; more precisely on the incremental likelihood ratio of the *t* value under the null and alternative hypothesis. To compute the likelihood ratio, we considered as the null hypothesis that there is no difference in mean confidence ratings between stimuli with fast- and slow-error responses in the binary recognition task. As the alternative hypothesis, we specified that there is a difference in mean confidence ratings for fast and slow errors with effect size *d*_*z*_ = 0.3.

Following previous work on the error-speed effect (Starns et al., 2018; Voormann et al., 2021), we used a median split to categorize the errors from the binary recognition task as fast or slow. More specifically, for each participant we computed separate medians depending on response correctness (correct vs. incorrect) and stimulus type (target vs. lure), and we defined all errors with RTs smaller than the respective median to be fast errors. Additionally, we inverted confidence ratings for lures in such a way that for every stimulus type a “6” indicates a strong confidence towards the correct response and a “1” indicates a strong confidence towards the incorrect response. For more details on the sequential sampling procedure, see Appendix A.

## Results

### Data preparation

We excluded all warm-up trials from the analysis. Furthermore, as pre-registered and analogous to Starns et al. (2018), we excluded trials with responses faster than 400 ms (to keep the number of avoidable errors low) or slower than 8 s in binary recognition decisions.^4^ This resulted in an exclusion of 0.31% of total trials in Experiment 1 and 0.09% of total trials in Experiment 2.

### Sequential probability ratio *t*-tests

#### Experiment 1

**Fig. 4:**
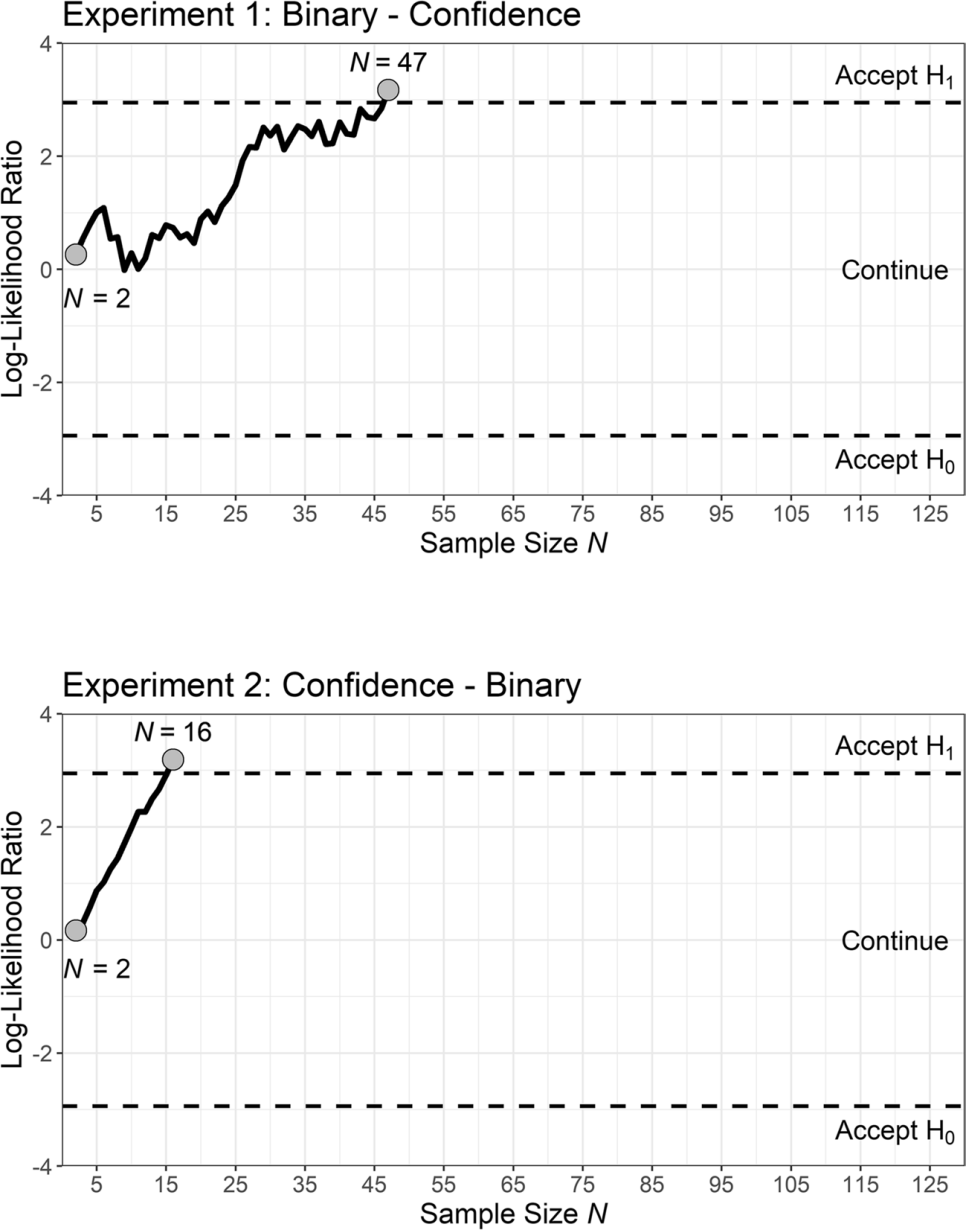
Sampling paths of the sequential probability ratio *t*-tests for the two experiments

On average, participants made errors in 27.8% of the trials during the binary recognition task. The mean RT of slow-error trials was 1,663 ms (*SD* = 678 ms) and the mean RT of fast-error trials was 834 ms (*SD* = 202 ms). Appendix B provides more extensive descriptive statistics for error and correct responses. After sampling 47 participants, the likelihood ratio exceeded the upper boundary indicating sufficient empirical evidence to accept the alternative hypothesis with 95% power (see Fig. 4), namely that there is a difference in confidence observed in a subsequent confidence-rating task for fast and slow errors of a preceding binary recognition task. As can been seen in Table 1, fast-error stimuli received on average more extreme confidence ratings in the direction of the incorrect response (i.e., values closer to 1) compared to slow-error stimuli. This effect is/was in line with the direction of the typical error-speed effect.

**Table 1:** Mean and standard deviation of the confidence ratings given to fast and slow errors of a binary recognition task as well as the results of the SPRT separately for Experiment 1 and 2. Smaller confidence values indicate a higher confidence towards the incorrect response

	Confidence fast error *M* (*SD*)	Confidence slow error *M* (*SD*)	*n*	*t*	LR
Experiment 1	2.76 (0.59)	2.92 (0.42)	47	2.67	23.89
Experiment 2	2.66 (0.55)	3.29 (0.64)	16	4.83	24.27

*Note*. The boundary to accept the alternative hypothesis amounted to A = 19 and the boundary for the null hypothesis amounted to B = .053

#### Experiment 2

Participants of Experiment 2 made an error in 30.1% of binary recognition trials on average. The mean RT of slow-error trials was 1,333 ms (*SD* = 315 ms) and the mean RT of fast-error trials was 735 ms (*SD* = 111 ms). Appendix B provides more extensive descriptive statistics for error and correct responses. After sampling 16 participants, the LR exceeded the upper boundary, indicating sufficient empirical evidence to accept the alternative hypothesis with 95% power (see Fig. 4), namely that there is a difference in confidence ratings between fast and slow errors. In accordance with Experiment 1 (see Table 1), fast-error stimuli received on average more extreme confidence ratings in the direction of the incorrect response compared to slow errors. Again, this effect corresponds to the pattern of the error-speed effect.

## Discussion

In Experiment 1, we replaced the 2AFC task that has so far been used as the second task in the error-speed paradigm by a confidence-rating task, and still found an analogue to the error-speed effect. More precisely, fast-error responses in the binary recognition task were associated with stronger confidence ratings towards these incorrect responses during the later confidence-rating task. Thus, error-speed effects seem to occur regardless of specific task characteristics or demands (such as the pairwise stimulus presentations in the usual 2AFC task), and independent of the type of measurement used (such as accuracy data in previous studies vs. confidence ratings in the present experiments). Replicating an error-speed effect using different tasks demonstrates that the effect reflects core mechanisms of recognition memory rather than being tied to one particular combination of tasks. The occurrence of this variant of the error-speed effect suggests that recognition errors result (at least partially) from probably quite stable misleading memory evidence. As outlined in the *Introduction*, models that incorporate such a notion – either directly, as in most continuous dynamic recognition models (e.g., diffusion model; Ratcliff, 1978), or from an additional discrete incorrect detection state (such as the 2LTM; Starns, 2021; Starns et al., 2018) – can account for the error-speed effect and its variants introduced in the present work. However, models that attribute all error responses to the same discrete mental state (such as the 2HTM; Snodgrass & Corwin, 1988) cannot explain this pattern of results.

In Experiment 2, we went one step further and reversed the order of tasks so that participants first had to complete a confidence-rating task and only then a binary recognition task. Results revealed that there was an error-speed effect regardless of task sequence. In line with recent studies, in which the error-speed effect also occurred when the item from the binary task was paired with a target or a lure not previously tested (Akan et al., 2023; Yüvrük et al., 2023), this refutes an alternative explanation of the error-speed effect whereby subsequent responses are informed by RTs from the preceding binary recognition task. Such an RT strategy was, for example, predicted by early models for confidence ratings (Audley, 1960). Instead, RTs, response accuracy, and confidence seem to tap the same latent construct, that is, stable-in-time memory evidence, which is a necessary precondition for any error-speed effect to manifest.

Previous studies already revealed that RTs and confidence ratings are correlated (Weidemann & Kahana, 2016). However, other than in the present study, participants of these earlier studies usually indicated how confident they are in their binary recognition decision immediately after making it. Nevertheless, not many recognition-memory models can jointly account for RTs, accuracy, and confidence ratings. Most models focus on, for instance, RTs and accuracy or RTs and confidence ratings, both within one task. However, they do not account for the relationship between these different metrics across tasks. Therefore, we had to consider a combination of the classical diffusion model (for binary responses) and the RTCON2 (for confidence ratings) under the assumption that the drift rate and the position of the match distribution tap into the same latent construct, namely underlying memory evidence.

However, there is also a model that considers the combination of binary decisions and subsequent confidence ratings: the two-stage dynamic signal detection model (2DSD; Pleskac & Busemeyer, 2010). In this model, binary responses are determined by a classical diffusion process. The subsequent confidence ratings, on the other hand, are determined based on a continued accumulation of evidence until the confidence response is actually required. The final confidence in the binary response therefore reflects the amount of evidence sampled during the binary decision process (stage 1) *and* during the inter-judgment time between the binary decision and the confidence-rating response (stage 2; Pleskac & Busemeyer, 2010). Although this model is well able to explain many typical patterns observed for RTs, accuracy, and confidence ratings, it is difficult to apply it in the present case since a confidence-rating trial did not take place immediately after the respective binary recognition trial but with a lag of up to 36 trials. Furthermore, in Experiment 2, confidence ratings were given *before* a binary response was given. Nevertheless, the fundamental assumption of the 2DSD according to which binary RTs, accuracy, and confidence ratings reflect different aspects of the same latent construct is supported by the present findings and the occurrence of the error-speed effect.

Taken together, the present study demonstrates that the error-speed effect is likely not just due to an RT strategy whereby participants base a later recognition response on the speed with which they executed an earlier recognition response. Rather, our findings support the notion that the error-speed effect is caused by misleading memory evidence. Furthermore, we show that the effect generalizes from 2AFC tasks to confidence-rating tasks, which is in line with the idea that the binary recognition task, the confidence-rating task, and the 2AFC task measure the same construct: (occasionally misleading) memory evidence.

## Appendix A

### Calculation of the sequential ratio probability t-test

Before conducting the sequential probability ratio test, one needs to specify three parameters (see Schnuerch & Erdfelder, 2020):

1. The effect size (${d}_{z}$ or *d* depending on the type of test)
2. Alpha error $$\alpha$$
3. Test power $$1-\beta$$

### Computation of the likelihood ratio

The decision about the sampling process is determined by the size of the likelihood ratio. The likelihood ratio defines the ratio of the likelihood of the data given the ${H}_{1}$ relative to the likelihood of the data given the ${H}_{0}$. This can be defined as:

$$L{R}_{n}= \frac{dt({t}_{n}|d{f}_{n}, {\Delta }_{1})}{dt({t}_{n}|dn, {\Delta }_{0})}$$

where *dt* denotes the density of the *t*-distribution (which is in R accessible via dt()). To compute the respective density the empirical ${t}_{n}$ value for all *n* cases, the degrees of freedom ${df}_{n} = n-1$, and the non-centrality parameter ${\Delta }_{n}$ have to be specified.

The empirical *t* value can be determined in the one-sample case by

$$t\left({df}_{n}\right)= \frac{\overline{d}\cdot \surd n}{{\sigma }_{D}}$$

with the mean difference between two dependent samples $\overline{d }$ being defined as

$$\overline{d } = \frac{{\sum }_{i=1}^{n}{x}_{1i}-{x}_{2i}}{n}$$

and the standard deviation of differences ${\sigma }_{D}$ being

$${\sigma }_{D} =\sqrt{\frac{{\sum }_{i=1}^{n}{{((x}_{1i}-{x}_{2i})-\overline{d })}^{2} }{n-1}}$$

where ${x}_{1i}$ and ${x}_{2i}$ denote the respective individual test values of person *i*.

The non-centrality parameter ${\Delta }_{n}$ describes the shift of the *t*-distribution on the horizontal axis. It is determined for the one-sample *t*-test by

$${\Delta }_{n} = {d}_{z} \cdot \surd n$$

Thus, the non-centrality parameter changes after each observation by the factor $\surd n$. The non-centrality parameter under the ${H}_{0}$ is 0.

### Decision process

Based on the height of the likelihood ratio value, the decision rule is as follows:

1. $L{R}_{n}\ge A$ accept ${H}_{1}$ and reject ${H}_{0}$
2. $L{R}_{n}\le B$ accept ${H}_{0}$ and reject ${H}_{1}$
3. $$B < L{R}_{n} < A$$ continue sampling.

The boundaries *A* and *B* can be computed considering the $\alpha$-error and power with

$$A = \frac{1-\beta }{\alpha }$$ and $$B = \frac{\beta }{1-\alpha }$$.

## Appendix B

Tables 2 and 3 provide descriptive statistics separately for errors and correct responses, and slow and fast target and lure responses in Experiments 1 and 2. As we used a sequential sampling design, caution is required when interpreting these descriptive statistics in terms of effect sizes.

**Table 2:** Error responses: overall proportion of errors (PEs) as well as mean response times (RTs), mean confidence ratings (Conf.) and proportion of trials in which participants switched their response (resp. switches) separately for fast and slow responses for targets and lures in Experiments 1 and 2

		Fast responses			Slow responses		
	PE	RT in ms	Conf.	Resp. switches	RT in ms	Conf.	Resp. switches
**Experiment 1**							
Targets	.44	826	2.80	.23	1616	2.93	.22
Lures	.12	982	2.83	.29	1859	3.20	.33
**Experiment 2**							
Targets	.36	768	2.77	.21	1303	3.08	.34
Lures	.29	799	3.20	.36	1410	3.69	.54

**Table 3:** Correct responses: overall proportion correct (PC) as well as mean response times (RTs), mean confidence ratings (Conf.) and proportion of trials in which participants switched their response (resp. switches) separately for fast and slow responses for targets and lures in Experiments 1 and 2

		Fast responses			Slow responses		
	PC	RT in ms	Conf	Resp. Switches	RT in ms	Conf	Resp. Switches
**Experiment 1**							
Targets	.56	801	5.26	.10	1410	4.68	.19
Lures	.88	771	4.74	.09	1366	4.37	.18
**Experiment 2**							
Targets	.64	712	5.20	.07	1182	4.59	.18
Lures	.71	749	5.19	.04	1295	4.84	.12

## Footnotes

1 Please note, whenever we refer to fast errors, we mean errors that are faster than the participants’ median error RTs. They do not refer to what is meant by fast errors in the diffusion model literature, namely extremely fast error responses that solely result from response bias and sampling noise; and thus, they are not predictive of the memory performance in another memory task. Those latter errors are also sometimes called avoidable errors (Starns et al., 2018), as they would elicit a correct response if more memory evidence was sampled. However, errors that we focus on are so-called unavoidable errors. Their idiosyncratic drift rates point towards the incorrect response boundary and thus an incorrect response would have also occurred if more memory evidence had been sampled.

2 To ensure the feasibility of the sample size, we defined a priori the maximum number of collected data to be N = 122 per experiment. This corresponded to the capability to detect an effect of size dz = .30 with α = .05 and β = .05 if a non-sequential design had been employed. As pointed out by Martin Schnuerch, it is inadmissible to conduct a classical null-hypothesis significance test when the maximum number of collected data is reached as it inflates the Type-I error rate of the overall procedure. Instead, it is good practice to refrain from any inferential procedure and simply report the likelihood ratio as a measure of the statistical evidence provided by the data. However, both experiments reached a conclusion before the maximum sample size was obtained.

3 Like previous studies (Starns et al., 2018; Voormann et al., 2021), we implemented alternating blocks of confidence ratings and binary decisions instead of administering each task as a whole. This reduced the confound between the item position and response time in the binary recognition task, as responses tend to become faster to the end of a list.

4 Admittedly, these RT cutoffs can be considered arbitrary. We, therefore, conducted robustness checks by additionally analyzing our data with RT cutoffs of 200 ms and 8 s as well as of 200 ms and 6 s, which led to the same pattern of results.

## Declarations

### Open practices statement

We preregistered Experiments 1 (https://osf.io/r8e4p/) and 2 (https://osf.io/6zuj3/) on the Open Science Framework (OSF) prior to data collection. All materials to run the experiments, the data, and data analysis script can be retrieved from the OSF (https://osf.io/3qzwh/).
